# Genotoxic Assessment of Nutraceuticals Obtained from Agricultural Biowaste: Where Do We “*AMES*”?

**DOI:** 10.3390/antiox11061197

**Published:** 2022-06-18

**Authors:** Giorgia Musto, Valentina Laurenzi, Giuseppe Annunziata, Ettore Novellino, Mariano Stornaiuolo

**Affiliations:** 1 Department of Pharmacy, University of Naples Federico II, Via Montesano 49, 80131 Naples, Italy; giorgia.musto@unina.it (G.M.); valentina.laurenzi95@gmail.com (V.L.); 2 Department of Medicine and Surgery, Università Cattolica del Sacro Cuore, 00168 Rome, Italy; ettore.novellino@unicatt.it

**Keywords:** recycling, biowaste, agricultural by-products, genotoxicity, nutraceuticals, circular economy, risk-assessment, Ames test, micronucleus test

## Abstract

Several pharmaceutical companies are nowadays considering the use of agri-food waste as alternative raw material for the extraction of bioactive compounds to include in nutraceuticals and food supplements. This recycling activity is encountering the support of authorities, which are alarmed by air, soil and water pollution generated by agricultural waste disposal. Waste reuse has several economic advantages: (i) its low cost; (ii) its abundance; (iii) the high content of bioactive molecule (antioxidants, minerals, fibers, fatty acids); as well as (iv) the financial support received by governments eager to promote eco-compatible and pollution-reducing practices. While nutraceuticals produced from biowaste are becoming popular, products that have been risk-assessed in terms of safety are quite rare. This despite waste biomass, in virtue of its chemical complexity, could, in many cases, mine the overall safety of the final nutraceutical product. In this review, we summarize the scientific results published on genotoxicity risk-assessment of bioactive compounds extracted from agricultural waste. The review depicts a scenario where the risk-assessment of biowaste derived products is still scarcely diffuse, but when available, it confirms the safety of these products, and lets us envisage their future inclusion in the list of botanicals allowed for formulation intended for human consumption.

## 1. Agri-Food By-Products as Raw Material for the Food/Nutraceutical Industry

### 1.1. Biowaste, a Contemporary Global Concern

Since 1960, modern and developed western societies have fully committed themselves to an unconscious consumeristic habit. Without a real need, capitalism has let people crave unnecessary amounts of food and food products, ultimately fostering the deleterious environmental impact caused by this massive request [1]. Along with the impact generated by food production (i.e., factory intensive livestock, deforesting for farming, river diversion), the environment suffers enormously from the pollution generated by the agri-food chain waste (biowaste), its improper handling and disposal [1,2,3,4,5,6].

Biowaste reaches, globally, a billion tons per year, and it has been estimated that more than 70% in weight of the starting biological material is disposed of. Biowaste is mainly composed by unprocessed agricultural by-products (organic materials, suspended solids, nitrogenous compounds, oils and grease) as well as by 2nd-best food products, whose exterior appearance does not fulfil the aesthetic requirement imposed by the globalized modern market [3,6]. Biowaste is endowed with high biological instability and high organic loading. When improperly disposed of, biowaste becomes the first cause of air (CO_2_, methane, ammonia and Nitric Oxide), water (chemicals like pesticides, fungicides and metals), microbes (bacteria, protozoa and viruses) and soil pollution [7,8].

The World Health Organization considers waste minimization as a mandatory task for modern society. In 2014, the European Food Safety Authority (EFSA) referred to reduction of biowaste pollution as a necessary global community effort, to be achieved thanks to the development of harmonized eco-compatible procedures for waste disposal and recycling [2,4].

While the recycling of waste biomasses for production of animal feed and biofuel [9,10] is nowadays a diffuse practice in many industrialized countries, the use of biowaste for the extraction of bioactive molecules to be included into products intended for human consumption (food supplements, novel/functional foods, and nutraceutical products) has recently drawn interest. In recent decades, the pharmaceutical industry has indeed proven the environmental and economic advantages of extracting biomolecules from biowaste [11]. The most fitting examples are represented by polyphenols, dietary fibers, peptides and lipids. Polyphenols, the biggest class of food-derived bioactive compounds included in food supplements, have historically known beneficial effects on human health [12,13,14,15], mostly due to their antioxidant activity. Such compounds are highly present in the portion immediately close to the plant epicarp, where they protect the plant against UV radiations, pathogens attack and physical damages [14,16,17]. Dietary fibers include: (i) soluble fibers (like β-glucans), fermented by the microbiota, that in turn produces health promoting metabolites like short-chain fatty acids [18]; (ii) and insoluble fibers (such as lignin, cellulose and hemicellulose) that bind bile acids in the intestine, reducing serum cholesterol and absorb and hold water, promoting intestinal transit [19]. Proteins and peptides from waste are being used as source of amino acids to include in food supplements [20]. Finally, fat residues of agricultural waste are an important source of omega-3 fatty acids eicosapentaenoic and docosahexaenoic acid, largely used as food supplements and endowed with scientifically proven beneficial health effects [21].

Considering the low cost of agricultural biowaste and the high amount of contained biomolecules, it is easy to understand why an increasing number of pharmaceutical companies are investing in biowaste recycling and why an equal number of scientific publications describe recycling procedures, as well as composition, health benefits of final products obtained from biowaste [22,23].

### 1.2. State-of-Art on the Risk-Assessment of Biowaste

While the scientific community should keep on fostering research on biowaste reuse, an issue on the safety of biowaste should necessarily remain open. Are biowaste-derived products safe for humans?

EFSA, at the light of the consultations of the panels Biological Hazard (BIOHAZ) [24] and Animal Health and Welfare (AHAW) [7], expressed its opinion and defined biowaste as a possible driver of emerging biological risk for the public health. The deriving *monitus* of this comment is that the obvious long-term environmental benefit of biowaste recycling could be undermined by its improper or incomplete risk-assessment.

Regarding the reuse of biowaste for nutraceutical formulations, two aspects of biowaste cast doubts on the safety of its derived products and their use for human consumption. The first relates to the chemical complexity of the biowaste. The technology used to process this material (often harsher as a consequence of the hardness of the plants’ parts used) might cause the release in the final product of chemical species potentially harmful to human health. Similarly, the use of the non-edible parts of the plant (roots, leaves, bagasse) could expose the consumers to toxins produced either by yeast or fungi, like aflatoxins B1. Environmental pollutants originating from pesticides and fertilizers could also represent the source of genotoxic compounds (polycyclic aromatic hydrocarbons, aromatic amines, quinolines, pyridines, nitroquinolines and hydroquinone) [25]. Finally, genotoxic elements, like heavy metals, can be often found in polluted irrigating waters [25].

The second concern about biowaste reuse relates to the eventual change in the absorption, distribution, metabolism, and excretion (ADME) profile of the final product that cannot in any way be considered identical to that obtained by traditional non-biowaste sources. Unexpected molecules could influence, synergistically, the bioactive fraction of the plant and alter the overall pharmacokinetic parameters of the final product (bioavailability, bioaccessibility, bioactivity) as well as its overall toxicity.

### 1.3. Tests for Genotoxic Risk-Assessment

In 2009, the EFSA published the guideline entitled “Safety assessment of botanicals and botanical preparations intended for use as ingredients in food supplements” [26,27]. The risk-assessment approach includes in vitro and in vivo experimental platforms organized into core areas of progressing complexity and economical effort [5,28,29,30]. The first, easiest and cheapest in vitro platform includes the assessment of genotoxicity. A substance is defined as genotoxic when it alters the structure of chromosomes, the information they contain or their way to properly segregate during cell mitosis. Genotoxic molecules can induce point mutations by chemically modifying one of the nitrogen bases or by reducing the proof-reading activity of human DNA polymerases. They can as well inhibit the correct segregation of sister chromatids during mitosis and cause chromosomal brakes and aneuploidy. Mutagenicity of food supplements is tested by means of the Ames test (Organization for Economic Cooperation and Development (OECD) guideline 471) [31] and the in vitro micronucleus test MNvit (OECD guideline 487) [32]. Alternative tests like the Comet Assay and the chromosomal aberration assay (CAA) can be as well conducted to exclude genotoxicity of the products. The same assessment and tests are suggested and requested by other regulatory advisory bodies (e.g., European Chemicals Agency [33]; UK Committee on Mutagenicity [34]; the US Environmental Protection Agency [35]).

#### 1.3.1. The Ames Test

The Ames test [36] identifies and measures mutations occurring in bacterial genome upon exposure to a test chemical. DNA damage can be considered a surrogate endpoint for carcinogenicity, since the latter occurs in mammals as a consequence of the accumulation of mutations [30]. The test exposes five bacterial strains (*Salmonella Typhimurium* and *Escherichia Coli*) to a test substance. The bacterial strains used in the Ames test present mutations in genes needed for the synthesis of a specific amino acid (His in *Salmonella Typhimurium* and Trp in *Escherichia Coli*). Differently from wild-type (wt) strains, these bacteria have lost auxotrophy for those amino acids and cannot grow in their absence [37]. Genotoxic chemicals revert the mutated gene sequences of the bacterial strains to the wt sequence allowing the growth of revertant colonies in the absence of amino acids. To identify chemicals acquiring genotoxicity upon in vivo metabolization, substances are tested with and without a metabolic activation system derived from rodent liver microsomes and referred to as S9 [38].

#### 1.3.2. The In Vitro Micronucleus Test (MNvit)

The purpose of the MNvit test [39] is to identify substances that cause structural and numerical chromosomal alteration in cells that have undergone cell division during or after the exposure to the test substance. The assay detects micronuclei in the cytoplasm of interphase cells and typically employs human or rodent cells lines and sometimes primary cell cultures. The MNvit test can be conducted in the presence or in the absence of S9 as well as of cytochalasin B, which is used to block cell division and generate binucleate cells. The MNvit allows an easy discrimination between genotoxic substances acting as clastogens or aneugens.

#### 1.3.3. Other Tests for Genotoxic Risk-Assessment

The single cell gel electrophoresis test, also known as Comet assay (OECD guideline 489) [40], is a simple test used to detect single or double-stranded DNA breaks after treatment with a potentially genotoxic substance. The test, that can be conducted both in vivo and in vitro, consists in loading cells on agarose gel between microscopic slides, to then lyse them using detergents and high salt solutions. A fluorescent dye is then used to stain DNA (such as ethidium bromide). Undamaged nuclei will appear as a round and homogeneous structures under the microscope; on the contrary, damaged cells will show tailored nuclei as consequence of DNA breaks and/or DNA damage.

Like the MNvit, chromosomal aberration assay (CAA) is used to identify substances that may cause structural chromosomal and chromatid aberration in metaphase cells. The test can be conducted both in vivo and in vitro, according to the OECD 475 [41] and OECD 473 [42] guidelines, respectively. For both tests, it is necessary to use a metaphase-arresting agent. In the in vitro version, a source of metabolic activation should be used, while the in vivo version is performed on rodents directly treated with the potentially genotoxic substances.

## 2. Genotoxic Risk-Assessment of Food Waste

While the rationale behind the EFSA’s suggestion to confirm the safety of nutraceutical products and the risk-assessment procedures is clear, how many products obtained from biowaste and presented by the scientific community as intended for human consumption have been risk-assessed?

In this review, we collect and discuss manuscripts (full and short articles and letters) reporting risk-assessment of biowaste products. In order to identify products that underwent at least the first group of in vitro tests suggested by EFSA, we focused on genotoxicity risk-assessment. The search of PubMed Medline (performed in May 2022) with the keywords “food waste (OR by-products, leaves, roots, stalks, bark, bagasse, straw residues, seeds, and woods)” AND “genotoxicity (OR mutagenicity, AMES, mutation, comet assay, micronuclei)” retrieved more than 150 results describing products obtained from agricultural waste and destined for human consumption, of which only 24 (Table 1) present experimental data on genotoxic risk-assessment. Of course, we cannot exclude that a different set of keywords would have retrieved more manuscripts, and we thus apologize to those authors that we have involuntarily not included in the review. Five publications are from a laboratory settled in Asia, where, probably, the elevated population further complicates biowaste management, and eight from Europe. As expected, the genotoxic risk-assessment was mainly performed on biowaste originating from highly consumed agricultural products like oil, tea, rice, wine and fruits, the ones more contributing to biowaste derived pollution.

### 2.1. Oil Biowaste

Palm oil, the main cooking oil in non-European countries, can be obtained from the fruits of several species of palms including *Laeis guineensis* and *Moringa oleifera*. Its production generates a large amount of waste (corresponding to 90% of the harvested fruits) and represents a serious environmental concern. Among palm oil by-products, the black liquor waste (BLW) contains lignin, a bioactive compound with health-promoting properties including antimicrobial, anti-parasitic, antioxidant, anticancer, genoprotective and immune protective activities [43], that gained the interest of food and pharmaceutical industries. Naik and co-workers [44] tested by MNvit the genotoxicity of lignin extracted from BLW (doses up to 200 mg/kg) administered for 2 days to Swiss albino mice. The authors did not observe genotoxicity and, on the contrary, report a genoprotective effect of BLW against mutations promoted in animals by cyclophosphamide.

Like palm oil, olive oil production generates by-products, the accumulation of which may represent a source of pollution and are of interest for pharmaceutical industries. *Olea Europea* cultivation occupies about 10 million hectares worldwide. Food industries mainly use its fruits, while leaves and flowers are not commonly used, although they present high amounts of phenolic acids, anthocyanin and flavonoids [45]. Kouka and colleagues [46] analyzed an olive blossom extract (containing among its components oleuropein and rutin) showing its non-genotoxicity using the Ames test (up to 32 mg per plate, on the *Salmonella Typhimurium* strain TA102).

In addition to leaves and flowers, three other types of waste are produced during olive oil manufacturing processes: olive mill wastewater, alperujo, and olive brine. These by-products contain several bioactive compounds including hydroxytyrosol, a compound endowed with antioxidant activity [47]. The genotoxicity of biowaste from olive mill wastewater was evaluated by the Ames test, using two of the five bacterial strains suggested by EFSA (*Salmonella* *Typhimurium* strains TA98 and TA100) with negative results registered up to 500 µg of Gallic Acid equivalents/plate [48]. Similarly, non-genotoxicity was confirmed for an hydroxytyrosol-enriched extract from mill wastewater assessed by both Ames test (*Salmonella* *Typhimurium* strain TA100 up to 5.0 µL per plate) and CAA test [49]. Alperujo, the other major waste from oil industry, is rich in hydroxytyrosol, verbacoside and tyrosol that have been reported to exert antioxidant, anti-inflammatory and antimicrobial activities. Anter and coworkers evaluated the genotoxic potential of this by-product performing a somatic mutation and recombination test on *Drosphila Melanogaster* wings. The product was not shown to be genotoxic up to the doses of 30 µL/mL [50].

Nonedible oil can also produce biowaste. The commonly used parts of *Juniperus communis* are berries and essential oil. The essential oil production generates a by-product known as post distillation water. Such a by-product is rich in polyphenols, including rutin, quinic acid, catechin and epicatechin. Vasilijević and colleagues [51] evaluated the genotoxic potential of this by-product on cancer and normal cells using the comet assay, observing that the waste product was genotoxic, especially on cancer cells.

### 2.2. Tea Biowaste

The second most drunk beverage in the world after water, tea, has a waste production reaching about the 4% of the entire production. The beverage is obtained by infusion of *Camellia sinensis* leaves, while flowers (despite them being rich in bioactive substances such as catechins, polysaccharides, flavonoids, and aminoacids) are wasted [52]. Tea biowaste has been shown to be non-genotoxic by Ames test (*Salmonella Typhimurium* strains TA97, TA98, TA100 and TA102), both in the absence or in the presence of S9 metabolic activation up to the doses of 5.0 mg/plate [53].

### 2.3. Rice Biowaste

Rice is one of the most consumed food worldwide. Its processing produces rice hush as biowaste, that represent about 20% of the rice processed. Thanks to its high content of phenolic (mainly anthocyanin) and non-phenolic compounds, numerous properties have been attributed to rice husk, including anti-inflammatory, antioxidants, anticancer activities [54]. Rice husk has been shown to be non-genotoxic (Ames test on *Salmonella Typhimurium* TA98 and TA100 strains) both in the presence and in the absence of metabolic activation by S9 at doses up to 5 mg [55,56].

### 2.4. Fruit and Nuts Biowaste

Citrus are the most widespread and consumed fruits in the world. Their production results in a large amount of waste, mostly peels, highly enriched in bioactive compounds. As consequence of the low cost of these wastes, the interest of industries for these by-products has recently grown. The genotoxicity of citrus peels (lemon, grapefruit, and mandarin) was tested on mouse splenocytes through the MNvit test, with negative results [57]. Similarly, non-genotoxic effects were reported for peels of mandarin of the cultivar *Citrus reticulata* (rich in nobiletin and tangertin) by Ames test (*Salmonella Typhimurium* strains TA100, TA98, TA1535, TA1537 and *Escherichia Coli* at the dose of 5 mg/plate) and MNvit tests [58]. A minimal genotoxicity was observed performing the chromosomal aberration test.

Like citrus, apples are highly appreciated and consumed fruits in the world, and possess several health-promoting properties, including prevention of cardiovascular and chronic diseases [59,60]. The cultivation of apples is widespread in the Mediterranean area and in particular in Italy. Apples contain dietary fibers (mainly pectin) and polyphenols (i.e., catechins, quercetin, procyanidins, phlorizin, rutin), mostly in peel [61,62] that, however, is considered a by-product of this cultivation. The genotoxicity of apple peel was assessed by the Comet assay test [63] and by the Ames test (*Salmonella Typhimurium* TA100, TA98, TA1537, TA1535 and *Escherichia* *Coli* doses up to 5 mg/plate), in both cases giving negative results [64].

Avocado is largely consumed in Latin America. Due to its abundance in phytosterols, triterpenes, fatty acids, and two new glucosides of abscisic acid, various beneficial effects have been referred to the consumption of this fruit, including antioxidant, antihypertensive and hypolipidemic properties [65]. Interestingly, avocado seed, a biowaste of this fruit, is considered an important raw material for the preparation of functional food and nutraceuticals. Camberos and colleagues [66] assessed the genotoxicity of Avocado seed through micronucleus tests, reporting negative results.

*Cyphomandra betacea* also known as tamarillo, or tree tomato, usually grows in south America and New Zealand. The ripe fruits are commonly used in the food field in the preparation of dishes. Ordóñez and colleagues confirmed the non-genotoxicity of an insoluble fraction of tamarillo pomace by the Ames test (*Salmonella Typhimurium* TA100, TA98 in the presence and in the absence of S9 metabolic activation) [67].

*Lansium domesticum* is a widely diffused fruit in Thailand, where is known as long-kong. However, only the pulp is consumed, while peel and seeds are wasted despite their high content of scopoletin, chlorogenic acid and rutin. The genotoxicity of this biowaste was assessed by Comet assay by Klungsupya and coworkers [68], who confirmed the non-genotoxic and, on the contrary, a mild genoprotective activity of this product.

An extract of Pomegranate waste (*Punica granatum L.*), mainly composed by the peel of the fruit, enriched in tannins (punicalagin, punicalin, elagic acid, and gallic acid) and flavanoids (catechin, anthocyanin, and other complex flavanoids) was shown non-genotoxic and genoprotective by MNvit and CAA assay [69].

Pecan nut shell is an important source of bioactive compounds, including tannins and other polyphenols [70]. This biowaste was found to be not genotoxic through the MNvit test [71].

### 2.5. Spice Biowaste

Saffron (*Crocus Sativus*) is a well-known and expensive spice that is obtained from dried stigmatic lobes of the pistil. The production of saffron results in a large production of waste materials. Of particular interest are the petals and anthers. These parts of the flowers, indeed, are rich in substances such as crocin and kaempferol, useful for the preparation of functional food and cosmetics [72]. Furthermore, antinociceptive, antimicrobial and antioxidant properties have been attributed to these by-products [73]. Chichiriccò and colleagues evaluated the genotoxicity of such biowaste through the MNvit test reporting negative results [74].

### 2.6. Winemaking Biowaste

Winemaking industry generates a large amount of waste (mainly grape pomace and stalks), that still results rich in bioactive compounds, mainly polyphenols, including stilbenes, phenolic acids, flavonoids, tannins and oligomeric procyanidins [75]. The absence of genotoxicity of a grape pomace extract destined for human consumption was shown non genotoxic by Badolati and colleagues using the Ames test (*S. Tiphymurium* TA100, TA98, TA1537, TA1535 and *Escherichia* *coli* doses up to 5 mg/plate) [64]. Grape seeds were shown to be non-genotoxic by MNvit up to 200 μg/mL [76]. Grape pomace has been as well shown to possess antigenotoxic proprieties [77].

### 2.7. Soybean Biowaste

United States are one of the largest producers of soybeans (around 32% of the world production) and fulfill the growing demand for this agricultural product. Such huge production is accompanied by the accumulation of by-products that are usually discarded despite being rich in bioactive compound (mainly isoflavones) [78]. Plewa and colleagues evaluated the genotoxic potential of soybean by-products trough the Comet assay, obtaining negative results. Moreover, the authors observed that soy biowaste is endowed with genoprotective activities and reduces damages induced by carcinogenic substances such as 2-acetoxyacetylaminofluorene.

### 2.8. Onion Biowaste

Onion is one of the most extensive crops in Europe and England and presents an associated waste production of about 50,000 tons per year. Onion by-products are rich in fructo-oligosaccharides with potential antioxidant and prebiotic proprieties. This biowaste was found to be non-genotoxic by the Comet assay on rat’s liver and leucocytes [79].

**Table 1:** Studies assessing the genotoxicity of bioactive fractions obtained using biowaste as a raw starting material.

Food (Waste By-Product)	Test Used	Experimental Model	Main Results		Ref.
			Genotoxic	Genoprotective	
Olive Oil (blossoms)	AMES	*Salmonella Typhimurium*	NO	YES	[46]
Olive Oil (Alperujo)	somatic mutation recombination test	*Drosophila* *Melanogaster*	NO	n.e.	[50]
Olive Oil (Raw olive mill waste water, Alperujo and olive brine)	AMES	*Salmonella Typhimurium* *Escherichia coli*	NO	n.e.	[48]
	CAA	Mammalian cells	NO	n.e.	
Olive Oil (Raw olive mill waste water)	AMES	*Salmonella Typhimurium* *Escherichia coli*	NO	n.e.	[49]
	CAA	Mammalian cells	NO	n.e.	
Palm Oil (black liquor waste)	MNvit	*Mus musculus*	NO	YES	[44]
Pecan Nut (shells)	MNvit	*Rattus norvegicus*	NO	YES	[71]
Tea (Flowers)	AMES	*Salmonella Typhimurium*	NO	n.e.	[53]
Tamarillo (pomace)	AMES	*Salmonella Typhimurium*	NO	n.e.	[67]
Saffron (anthers and tepals)	MNvit	Mammalian cells	NO	n.e.	[74]
Onion (by-product)	Comet assay	*Rattus norvegicus*	NO	n.e.	[79]
Soy beans (by-product)	Comet Assay	Mammalian cells	NO	YES	[80]
Apple (peel)	Comet assay	Mammalian cells	NO	YES	[63]
Apple (pomace)	AMES	*Salmonella Typhimurium* *Escherichia Coli*	NO	n.e.	[64]
Long-kong (peel)	Comet Assay	Mammalian cells	NO	YES	[68]
Hawthorn (leaf and bark)	MNvit	*Murine model*	YES	n.e.	[81]
Rice (husk)	AMES	*Salmonella Typhimurium*	NO	n.e.	[55,56]
	MNvit	*Rattus norvegicus*	NO	n.e.	
Juniper (post distillation water)	Comet assay	Mammalian cells	YES	n.e.	[51]
Avocado (Seed)	MNvit	*Mus musculus*	NO	YES	[65]
Mandarin (peel)	MNvit	*Mus musculus*	NO	n.e.	[58]
	CAA	Mammalian cells	YES	YES	
	AMES	*Salmonella Typhimurium* *Escherichia Coli*	NO	n.e.	
Citrus fuits (peels)	CAA	*Mus musculus*	NO	n.e.	[57]
Grapes (grape pomace)	AMES	*Salmonella Typhimurium* *Escherichia Coli*	NO	n.e.	[64]
Grapes (grape pomace)	Comet assay	Mammalian cells	NO	YES	[77]
Grapes (seeds)	MNvit	Mammalian cells	NO	YES	[76]
Pomegranade (peel)	CAA	*Mus musculus*	NO	YES	[69]

Abbreviations: CAA: chromosomal aberration test MNvit in vitro micronucleus test; n.e., not evaluated.

### 2.9. Hawthorn Biowaste

*Crataegus oxyacantha L*. is a deciduous tree member of Rosaceae family commonly known as Hawthorn. The uses of many parts of the plant, i.e., leaves, flowers, and unripe berries have been described for their traditional use (e.g., astringent, antispasmodic, cardiotonic, diuretic, hypotensive, and anti-atherosclerotic). Recently, the barks, one of the by-products of its processing, are under investigation for their nutraceutical properties. Aguilera-Rodríguez and colleagues [72] evaluated the genotoxicity of both aqueous and hydroalcoholic extracts of the bark by MNvit test. The bark aqueous extract presented dose-depending short-term and long-term genotoxicity. The hydroalcoholic bark extract showed only genotoxic long-term effects.

## 3. Conclusions and Future Perspectives

As a consequence of the expected increase in the world population [82], the production of food and biological products in the next decades will have an enormous impact on the ecosystem of our planet [1]. Among the different forms of agri-food-related pollution, the disposal of biowaste from agricultural productions will represent a serious calamity for air, lands and waters. Modern society is moving towards the reduction of biowaste, also thanks to the promotion of its recycling. In order to foster the recycling of biomasses and lower their environmental impact, governments and international authorities are financing public entities (Universities and Research Institutes) as well as private companies investing in waste recycling. Among the other recycling activities, agri-food biowaste is being used as starting raw materials for sustainable production of nutraceuticals, food supplements and fortified foods.

Aligned to the effort of the general community, science is contributing to this global mission by fostering research aimed at the valorization of biowaste, including its reuse for pharmaceuticals and nutraceuticals purposes. Among the scientific issues, and as suggested by authorities like EFSA, science must address the safety of biowaste reuse. We have here reviewed scientific articles describing risk-assessment of products obtained from biowaste and intended for human consumption. At the end of this survey, we can conclude that compared to the number of nutraceuticals produced form waste, still too few have been risk-assessed.

Genotoxic risk-assessment of bioproduct obtained from agricultural waste has to represent, of course, only a first step in the confirmation of the their safety [83]. The risk-assessment should be completed and include all the toxicological studies requested by the authorities. Similarly, the hygiene of the final products must be confirmed. The product obtained from waste should not contain microbial contamination. Pathogens colonizing the waste and the associated infectious diseases may be spread from biowaste to animals and humans. Similarly, the absence of microbial byproducts (toxins, allergens), heavy metals and organic contaminants should also be proven. The presence of antibiotics in the recycled biowaste should be carefully checked because it could contribute to the spread of antibiotic-resistant micro-organisms. The quality of biowaste is thus of enormous importance and must be guaranteed so that society could fully benefit from waste recycling without any risk for humans, animals and for the environment itself [84].

The lack of safety confirmation for food products obtained from waste is very risky and might mine the overall market and the investments that industries are making in this important recycling practice. The public of consumers is acquiring a growing culture on the health properties of natural products (mostly dispensed by web and social media). Consumers are nowadays aware that the paradigm “*biological = safe*” is not an ultimate truth and pretend that the products they consume are safe. They are supported by authorities, that, from a regulatory point of view, advise developers and producers to act responsibly and confirm the safety of their products by showing their compliance with the safety requirements set out in the general food law. Despite this advice, the lack of a firm regulation has allowed the setting of a “foggy landscape” in which developers and producers of biowaste-derived products move autonomously. Many companies dispense themselves from risk-assessment as they use as starting material the biowaste of plants included in the national compendium of allowed botanicals. However, in the case of biowaste, the lists do not clearly cite biowaste among the biomass to include in the final products. More importantly, in case of an incurring safety issue (intoxication or unexpected side effects), the fact to have used products included in a list of allowed botanicals will not add any force to an eventual legal defense.

What could further convince companies to risk-assess is that, in the light of the results so far presented by the scientific literature (here reviewed), products obtained from biowaste are in most cases safe, at least in terms of genotoxicity. The biosafety of the extracts obtained from traditional sources indeed seems to also be maintained when the bioactive fractions are extracted from biowaste. Some of the extracts even present genoprotective activity against known mutagens. Producers and stakeholders could thus envisage a likely future scenario where the recycled biowaste will be allowed for the preparation of nutraceuticals and the practice of recycling is further supported.

## Data Availability

Not applicable.
